# Detecting Deoxyhemoglobin in Spinal Cord Vasculature of the Experimental Autoimmune Encephalomyelitis Mouse Model of Multiple Sclerosis Using Susceptibility MRI and Hyperoxygenation

**DOI:** 10.1371/journal.pone.0127033

**Published:** 2015-05-18

**Authors:** Nabeela Nathoo, James A. Rogers, V. Wee Yong, Jeff F. Dunn

**Affiliations:** 1 Hotchkiss Brain Institute, University of Calgary, Calgary, Alberta, Canada; 2 Department of Radiology, University of Calgary, Calgary, Alberta, Canada; 3 Department of Clinical Neurosciences and Oncology, University of Calgary, Calgary, Alberta, Canada; 4 Experimental Imaging Centre, University of Calgary, Calgary, Alberta, Canada; Friedrich-Alexander University Erlangen, GERMANY

## Abstract

Susceptibility-weighted imaging (SWI) detects hypointensities due to iron deposition and deoxyhemoglobin. Previously it was shown that SWI detects hypointensities in the experimental autoimmune encephalomyelitis (EAE) model of multiple sclerosis (MS), most of which are due to intravascular deoxyhemoglobin, with a small proportion being due to iron deposition in the central nervous system parenchyma and demyelination. However, animals had to be sacrificed to differentiate these two types of lesions which is impractical for time course studies or for human application. Here, we proposed altering the inspired oxygen concentration during imaging to identify deoxyhemoglobin-based hypointensities *in vivo*. SWI was performed on lumbar spinal cords of naive control and EAE mice using 30% O_2_ then 100% O2. Some mice were imaged using 30% O_2_, 100% O_2_ and after perfusion. Most SWI-visible hypointensities seen with 30% O_2_ changed in appearance upon administration of 100% O_2_, and were not visible after perfusion. That hypointensities changed with hyperoxygenation indicates that they were caused by deoxyhemoglobin. We show that increasing the inspired oxygen concentration identifies deoxyhemoglobin-based hypointensities *in vivo*. This could be applied in future studies to investigate the contribution of vascular-based hypointensities with SWI in EAE and MS over time.

## Introduction

Susceptibility-weighted imaging (SWI) is an MRI method that is sensitive to iron in the parenchyma, demyelination and deoxyhemoglobin [1, 2]. Due to its enhanced sensitivity to deoxyhemoglobin, SWI has been used to visualize the venous vasculature [3, 4]. Lesions are commonly detected in patients with multiple sclerosis (MS) with SWI [5, 6]. However, since SWI is sensitive to multiple elements (e.g. parenchymal iron deposition, demyelination, deoxyhemoglobin), one of the limitations in patient studies is the lack of specificity of the element(s) causing signal changes.

It is important to be able to identify SWI hypointensities due to deoxyhemoglobin in particular, in MS patients, as changes in blood oxygenation levels may be suggestive of a decoupling between the demand and supply of oxygen in tissue via reduced perfusion which has been shown to be present in MS patients [7, 8]. Reduced perfusion with the same or increased metabolic rate would lead to hypoxia, and this would appear as hypointense signal in susceptibility MRI. Alterations in blood oxygenation levels may also be related to changes in metabolism, which has also been shown to be present in the brains of MS patients [9].

In the chronic inflammatory model of MS, experimental autoimmune encephalomyelitis (EAE), SWI has detected hypointensities in the cerebellum and spinal cord, where many of these hypointensities were due to deoxyhemoglobin in the vasculature while a smaller number of hypointensities were due to parenchymal iron deposition [10]. However, in that study, MRI was carried out in animals that had been sacrificed (perfused) to identify deoxyhemoglobin-based hypointensities. This would not be practical in a clinical setting.

In order to differentiate hypointensities due to intravascular deoxyhemoglobin from those due to parenchymal iron deposition in patients, an *in vivo* method is required. In this study, we proposed a method whereby deoxyhemoglobin-based hypointensities could be identified *in vivo*. The visibility of the venous vasculature is highly dependent on the partial pressure of oxygen, and modulating oxygen has been used in conjunction with blood-oxygen level dependent (BOLD) MRI. However, modulating oxygen for identifying deoxyhemoglobin-based hypointensities has not been carried out in the context of MS.

The aim of this study was to determine if deoxyhemoglobin-based hypointensities could be identified in SWI images by imaging naïve control and EAE mice using two different oxygen concentrations: a standard inspired oxygen concentration (30%) and a high inspired oxygen concentration (100%). Our hypothesis was that vascular hypointensities visible with 30% O_2_ would disappear upon administration of 100% O_2_, as the alteration of oxygen levels would only affect the oxygenation of hemoglobin in the blood. This has been demonstrated in human studies where the level of inspired oxygen has been modulated [11]. Here, we show that we can identify hypointensities in SWI images caused by intravascular deoxyhemoglobin *in vivo*, providing an avenue for investigating the proportion of hypointensities due to deoxyhemoglobin in patient studies.

## Materials and Methods

### Mice and induction of EAE

Female C57BL/6 mice, six- to eight-weeks old, were acquired from Charles River (Montreal, Canada). Animal experiments were carried out in strict accordance with guidelines created by the Canadian Council on Animal Care. The protocol was approved by the Animal Care Committee at the University of Calgary. All EAE immunizations were performed under ketamine/xylazine anaesthesia, and all efforts were made to minimize suffering.

At 8–10 weeks of age, mice were immunized with EAE using methods described previously, which consisted of injecting 50 μg of myelin oligodendrocyte glycoprotein (MOG)_35–55_ peptide emulsified in complete Freund’s adjuvant and 10 mg/mL of heat-inactivated *Mycobacterium tuberculosis* subcutaneously in both hind flanks [12]. Mice received 300 ng of pertussis toxin via intraperitoneal injection on the same day as and two days after MOG immunization [12]. To assess motor disability, mice were scored using a 15-point grading scale whereby each limb and the tail were scored separately [13]. With this grading scale, the tail received a maximum score of two when completely paralyzed or a score of one when paretic. Each limb received a maximum score of up to three if fully paralyzed, a score of two if there was paresis, or a score of one if there was weakness or altered gait. A mouse that is fully paralyzed would receive a score of 14 and a score of 15 represents death.

### *In vivo* MRI acquisitions for different concentrations of oxygen in inhalation gas

For all MRI acquisitions, a 9.4T Bruker Avance console was used with a 20 mm diameter surface coil. Imaging was carried out on naive control mice (*n* = 9), EAE mice at peak disease, between days 15 and 19 post-injection (*n* = 9), and EAE mice at long-term disease, between days 31 and 45 post-injection (*n* = 5). Imaging was conducted halfway through T13 down to the early part of L5 of the spinal cord, covering the majority of the lumbar spinal cord. Images were acquired in the axial orientation. For imaging with 30% O_2_ and 100% O_2_, 3D gradient echo with flow compensation in all three directions was used. Imaging parameters were as follows: matrix = 192 x 128 x 32 (zero-filled to 256 x 128 x 32), FOV = 0.92cm x 1.28cm x 1.28cm, TE = 4ms, TR = 50ms, flip angle = 15°, averages = 17, and acquired voxel size = 48μm x 100μm x 400μm (displayed voxel size = 36μm x 100μm x 400μm), acquisition time = ~58 minutes. Mice were initially anaesthetized using 2.5–3% isoflurane and were kept anaesthetized throughout imaging using 1.7–2.2% isoflurane.

Mice were imaged first using a mixture of 30% O_2_/70% N_2_. At the end of this MRI acquisition, the gas was switched to 100% O_2_. To ensure a physiological equilibrium was reached after starting 100% O_2_, we waited 10 minutes before starting the second MRI acquisition. The same MRI acquisition was carried out with 100% O_2_ as was performed using the 30% O_2_/70% N_2_ mixture. As the mouse did not have to be moved during imaging, the images acquired using the two different percent oxygen concentrations could be directly compared. Isoflurane was adjusted as needed to ensure the mouse’s respiration rate remained consistent. The mouse’s temperature was monitored throughout imaging.

### Pre- and post-perfusion MRI acquisitions

A subset of mice from the naïve control group (*n* = 4) and the EAE group at long-term disease (*n* = 4), were imaged with 30% O_2_/70% N_2_, then 100% O_2_ as noted above. After the MRI acquisition with 100% O_2_, the animal was perfused. The animal was imaged after perfusion using methods described previously [10], with the same coil and imaging parameters as those used for the *in vivo* MRI acquisitions described above.

### MR image processing

Signal Processing in NMR (SPIN) software (MRI Institute, Detroit, MI) was used to process magnitude and phase MRI data. Image processing was carried out with methods described previously [14]. Phase data was filtered using a 32x32 Hanning filter to create filtered phase images, and a negative phase mask was multiplied into the magnitude data four times to create SWI images.

### Counting of hypointensities in SWI MRIs

Hypointensities were counted in blinded fashion by two researchers; values for the two researchers were averaged for analysis. Data were used from naïve mice (*n* = 9) and EAE mice at peak disease that had a behavior score of 6 or higher (*n* = 6). We only used animals with behavior scores above 6 because we wanted to ensure that we have a group of animals where we were confident that they would show hypointensities, and so we only used those with severe behavior changes. In our experience, there are more hypointensities when there is greater motor dysfunction. We set our criteria for motor function before carrying out the study. Since only 2/6 long-term EAE mice had behavior scores of 6 or higher, we did not include long-term EAE mice in our statistical analysis, but we have commented on this in the results. One additional peak EAE mouse was in the study, but was not included in the statistics. This animal showed an increase in hypointensities with hyperoxygenation. We believe this animal was becoming severely hypoxic due to the duration of the anesthesia, and so it was no longer in an acceptable physiological condition. Every fifth slice was used for counting for each spinal cord, and a total of five slices were counted per animal.

Images acquired with 30% O_2_ were put side-by-side with images acquired with 100% O_2_ for a given animal. The number of hypointensities seen with 30% O_2_ were counted and then compared with those that appeared different in the corresponding image acquired with 100% O_2_. Hypointensities that had been observed with 30% O_2_ data were classified using 100% O_2_ data into one of the following categories: 1) hypointensity seen with 30% O_2_ disappeared with 100% O_2_; 2) hypointensity appeared less hypointense with 100% O_2_ than was seen with 30% O_2_, but did not disappear completely; or 3) hypointensity seen with 30% O_2_ became hyperintense (bright) with 100% O_2_. Hypointensities observed that did not fall into one of these categories were assumed to remain the same in appearance with 30% O_2_ and 100% O_2_.

### Statistical analysis

A two factor univariate repeated measures analysis of variance (ANOVA) was carried out to compare if there was a significant difference in the number of hypointensities using the animal type (control vs. peak EAE) and oxygen level (30% O_2_ vs. 100% O_2_) as the two factors. If these factors were found to significantly affect the number of hypointensities, then individual comparisons were carried out as described below.

The number of hypointensities seen with 30% O_2_ between naive and peak EAE mice was compared using an independent *t*-test. To compare the number of hypointensities seen with 30% O_2_ with the number that remained unchanged upon administration of 100% O_2_, paired *t*-tests were used for naive control and peak EAE data. To compare the different response types observed with 100% O_2_ between control and EAE mice, independent *t*-tests were used for each response type.

To compare fluctuations in respiration rate and body temperature due to hyperoxygenation, paired *t*-tests were used to compare these parameters 15 minutes before switching to 100% O_2_ (while 30% O_2_ was still being used during imaging) and 15 minutes after switching to 100% O_2_. In instances where assumptions for normality or equal variance were violated, the non-parametric equivalent of the parametric test was used. For all statistical tests used, *p*≤0.05 was considered significant.

## Results

### Behavioral assessment

All mice were scored for behavior on the day of imaging except for one long-term EAE mouse (**S1 Table**). Naïve control mice (*n* = 9) all had scores of 0. A score of 0 indicates no deficit, while most EAE animals tend to have scores in the range of 6–10. Peak EAE mice (*n* = 9) had an average score of 5.1 ± 1.1 (mean ± SEM). Long-term EAE mice (*n* = 4) had an average score of 6.5 ± 1.2.

### Physiological parameters before and after hyperoxygenation

Data for respiration rate and body temperature was obtained in the majority of mice imaged (*n* = 20) (**S2 Table**); reliable data could not be obtained for three mice and so they were not included in the analysis. There was no significant difference in the average respiration rate (breaths/minute) with 30% O_2_ (99.8 ± 16.5, mean ± SEM) as compared to 100% O_2_ (100.0 ± 16.6) (*p* = 0.972, paired *t*-test). There was also no significant difference in the average temperature (in °C) with 30% O_2_ (36.64 ± 0.09, mean ± SEM) as compared to 100% O_2_ (36.66 ± 0.09) (*p* = 0.505, paired *t*-test).

### Responses to hyperoxygenation

**Fig 1:**
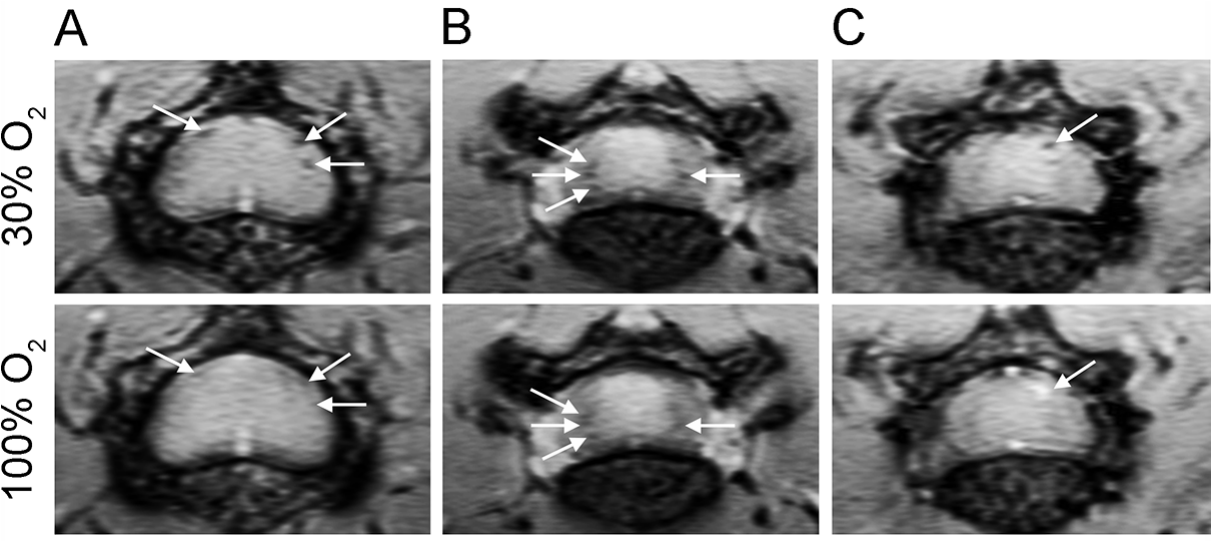
Deoxyhemoglobin-based SWI hypointensities in the lumbar spinal cords of EAE mice that appear hypointense with 30% O_2_ show various responses upon administration of 100% O_2_. (A) Some hypointensities seen with 30% O_**2**_ disappear with 100% O_**2**_ (white arrows). (B) Some hypointensities seen with 30% O_**2**_ become less dark with 100% O_**2**_, but do not disappear completely (white arrows). (C) Some hypointensities seen with 30% O_**2**_ become hyperintense with 100% O_**2**_ (white arrow).

We showed previously that SWI shows hypointensities that relate to deoxyhemoglobin in blood vessels in the lumbar spinal cords of EAE mice [10]. These hypointensities have been observed at the grey/white matter boundary of the spinal cord and in the spinal pia mater. With hyperoxygenation, it would be expected that deoxyhemoglobin concentration in the veins would decrease, and that hypointensities would become less dark. In fact, with hyperoxygenation, three different types of responses were observed. In the presence of 100% O_2_, hypointensities either: disappeared completely (**Fig 1A**); became less hypointense, but did not disappear completely (**Fig 1B**); or they became hyperintense (**Fig 1C**).

### Vascular hypointensities that alter in appearance with hyperoxygenation disappear after perfusion

**Fig 2:**
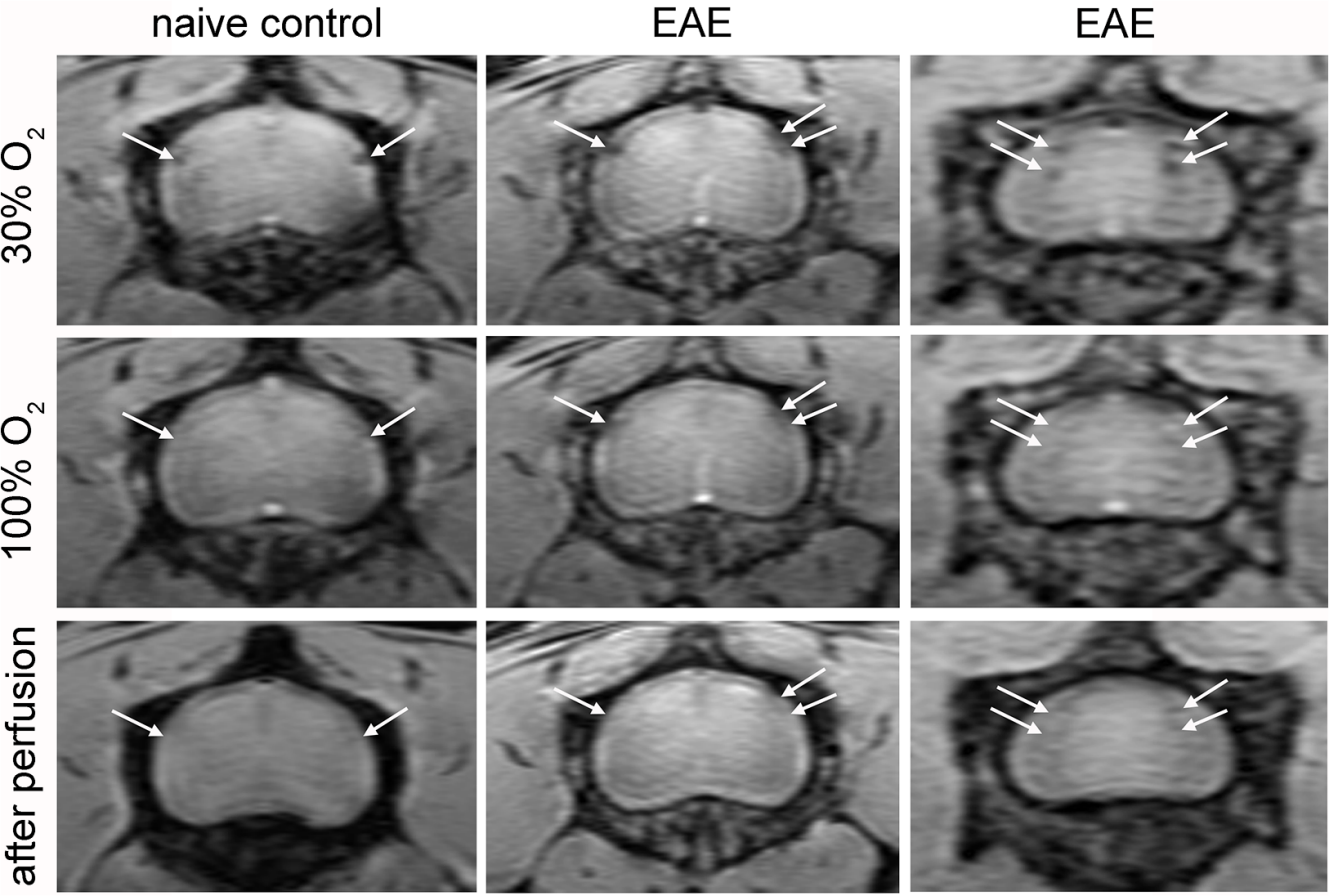
Hypointensities observed with 30% O_2_ that change in appearance with 100% O_2_ are not seen after perfusion. Left column: SWI MR images obtained in a naive control mouse where hypointensities visible with 30% O_**2**_ disappear with 100% O_**2**_ (white arrows). Middle and right columns: SWI MR images obtained in EAE mice, where hypointensities show different responses with 100% O_**2**_; some disappear after perfusion (white arrows). As all hypointensities which changed with 100% O_**2**_ disappear after perfusion, they are likely due to intravascular deoxyhemoglobin.

A subset of naive control and long-term EAE mice were imaged with 30% O_2_, 100% O_2_ and after perfusion, as the perfusion method had provided good visualization of hypointensities disappearing after perfusion which were due to deoxyhemoglobin [10]. Mice which underwent the three imaging acquisitions showed hypointensities with 30% O_2_ which changed in appearance with 100% O_2_ and disappeared after perfusion (**Fig 2**). This phenomenon was observed in both naïve control and EAE mice, and did not depend on what type of response was seen with 100% O_2_, so long as the appearance with 100% O_2_ was different from that seen with 30% O_2_. Taken together, this data supports that hypointensities which changed with hyperoxygenation were due to deoxyhemoglobin.

### Most hypointensities seen with 30% O_2_ change in appearance with 100% O_2_

For peak EAE mice included for counting (*n* = 6) and naïve control mice (*n* = 9) (**S3 Table**), analysis was first carried out to determine the effects of two factors on the number of hypointensities: animal type (control vs. peak EAE) and oxygen concentration (30% vs. 100%). It was found that both factors were significant (*p*<0.001 for each factor, two-factor univariate repeated measures ANOVA). This enabled for carrying out individual comparisons.

**Fig 3:**
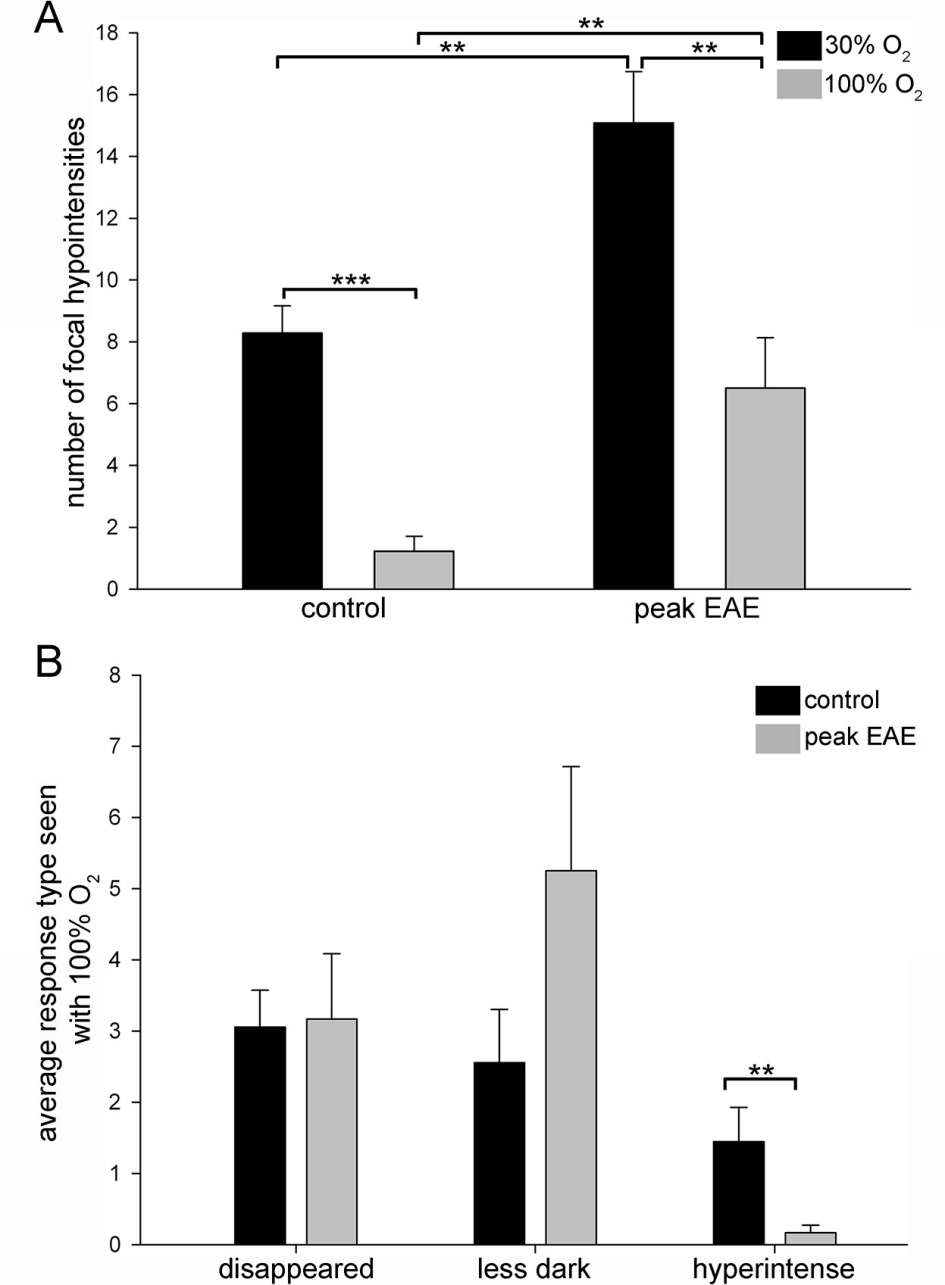
Quantitative assessment of responses of SWI hypointensities to hyperoxygenation. (A) The number of hypointensities seen before and after hyperoxygenation. Most hypointensities change with hyperoxygenation in both naïve controls and peak EAE mice. (B) Average response type seen with 100% O_**2**_, namely hypointensities disappearing, hypointensities becoming less dark but not disappearing completely and hypointensities becoming hyperintense. The number of hypointensities that became hyperintense upon administration of 100% O_**2**_ was significantly greater in naïve control mice than in peak EAE mice. **p<0.01 and ***p<0.001 by paired *t*-test in (A), **p<0.01 by Mann-Whitney U test in (B).

There was a significant difference in the number of focal hypointensities seen with 30% O_2_ between naive control (8.3 ± 0.9, mean ± SEM) and peak EAE mice (15.1 ± 1.7) (*p* = 0.002, independent *t*-test). The number of hypointensities seen with 30% O_2_ was significantly greater than the number of non-changing hypointensities (with 100% O_2_) in both naïve controls (*p*<0.001, paired *t*-test) and peak EAE mice (*p* = 0.009, paired *t*-test) (**Fig 3A**). Furthermore, the number of hypointensities that did not change in appearance with 100% O_2_ was significantly greater in peak EAE mice (6.5 ± 1.6) than in naïve controls (1.2 ± 0.5) (*p* = 0.003, independent *t*-test) (**Fig 3A**).

The breakdown of response types was also compared between naïve controls and peak EAE mice. The average number of hypointensities that disappeared in naïve controls (3.1 ± 0.5, mean ± SEM) and peak EAE mice (3.2 ± 0.9) was not significantly different between the two groups (*p* = 0.911, independent *t*-test). The average number of hypointensities that became less dark but did not disappear completely was also not significantly different between naïve controls (2.6 ± 0.7) and peak EAE mice (5.3 ± 1.5) (*p* = 0.095, independent *t*-test). However, the average number of hypointensities that became hyperintense was significantly different between naïve controls (1.4 ± 0.5) and peak EAE mice (0.2 ± 0.1) (*p* = 0.006, Mann Whitney U-test) (**Fig 3B**). Of note, only 2 of 6 peak EAE mice had hypointensities that became hyperintense, whereas all (9 of 9) controls had hypointensities that became hyperintense with 100% O_2_.

Of the total number of hypointensities seen with 30% O_2_ in naïve controls, 85.2% altered in appearance when 100% O_2_ was administered. Of these, 43.3% disappeared, 36.2% became less dark but did not disappear completely and 20.5% became hyperintense (**Table 1**). In peak EAE mice, 56.9% altered in appearance when 100% O_2_ was administered. Of these, 36.9% disappeared, 61.2% became less dark but did not disappear completely and 1.9% became hyperintense (**Table 1**).

**Table 1:** Proportion of response types to 100% O_2_ for all animals combined.

Animal type		Response type (%)	
	Disappear	Less dark	Hyperintense
Control	43.3	36.2	20.5
Peak EAE	36.9	61.2	1.9

### SWI hypointensity counting is correlated between two blinded researchers for both 30% O_2_ and 100% O_2_ data

To determine how consistent counting of hypointensities was between the two blinded researchers, Pearson’s correlation was used for the number of hypointensities seen with 30% O_2_ and the number of hypointensities that changed with 100% O_2_. There was a strong positive correlation for counting of hypointensities between the two researchers for data acquired with 30% O_2_ (r = 0.773, *p*<0.001, Pearson’s correlation), indicating that counting was consistent between individuals. For the number of hypointensities that changed with 100% O_2_, there was also a significant positive correlation between the two researchers (r = 0.574, *p* = 0.025, Pearson’s correlation). Taken together, these data indicate that the consistency of counting between researchers was acceptable.

## Discussion

In this study, we have shown that altering the inspired oxygen concentration in combination with *in vivo* SWI provides a means to identify hypointensities due to intravascular deoxyhemoglobin in the EAE model, which has not been done before. This method could be translated to the clinic for MS patients, where 100% O_2_ could be used to identify hypointensities due to deoxyhemoglobin with SWI. It is possible that adding CO_2_ as well as O_2_ would increase the sensitivity to changes, as this mixture also increases cerebral blood flow [15].

In EAE studies, 100% O_2_ has been used as part of the inhalation gas to differentiate microglia/macrophages containing iron nanoparticles from venous blood vessels [16, 17], but not as a means to focus on identifying hypointensities due to intravascular deoxyhemoglobin as we did in this study. It is important to identify hypointensities due to deoxyhemoglobin in EAE mice because we have found in this study and in our previous work [10] that the number of hypointensities detected with SWI is significantly elevated in the CNS of EAE mice compared to controls. We propose that this is due to venous hypoxia, where there may be increased demand for oxygen due to inflammation where supply cannot keep up (increased oxygen extraction).

As mentioned, most hypointensities observed were vascular and changed in the presence of 100% O_2_. Specifically, three different responses were observed: 1) hypointensities disappeared, 2) hypointensities became less dark but did not completely disappear, or 3) hypointensities became hyperintense. The cause of a conversion to hyperintensity is puzzling, as reductions in deoxyhemoglobin are not likely to change the relaxation times for blood to become bright. It is possible there is an out of volume effect related to flowing spins. Of note, one third of peak EAE animals had any hypointensities become hyperintense while all controls had hypointensities become hyperintense with 100% O_2_. One possible reason for these varying responses is that the vessels had different oxygen saturations to begin with. Lower oxygen saturation in spinal cord vessels of EAE mice would be suggestive of venous hypoxia in EAE. Another possibility is that some vessels in EAE mice had perivascular iron, but this is unlikely, as we did not observe SWI hypointensities after perfusion in the corresponding locations.

There were some hypointensities that did not change with 100% O_2_ in EAE mice. It is possible that this was due to those hypointensities having too low of an oxygen saturation to be affected by the hyperoxygenation administered during imaging. Another possibility is that these vessels are occluded somehow which prevents 100% O_2_ from having any effect on the vessels; such occlusion has been observed in EAE mouse spinal cord vessels using T_2_-weighted MRI [18].

It should be noted that, in this study, we did not see any obvious parenchymal lesions. That is, no lesions appeared in the white matter that could be attributed to a lesion using histopathology. Importantly, only hypointensities in the ventral white matter have been due to parenchymal iron/demyelination which we had published on previously. However, these were rarely seen [10]. It has been observed by others that, in general, models of MS have little parenchymal iron [19], but parenchymal iron is observed in MS [20] and is an important contributor to signal changes in susceptibility MRI in MS.

With respect to limitations, we observed that when switching from 30% O_2_ to 100% O_2_, the animal must be monitored closely as there is a chance for a significant change in respiration which may cause a shift in the position of the spinal cord. This may lead to imperfect co-localization between images obtained at 30% O_2_ and 100% O_2_.

An alternative to 100% O_2_ for altering the appearance of the venous vasculature is to include CO_2_, which acts to increase blood flow [21]. Carbogen (95% O_2_/5% CO_2_) has been used to reduce the contrast provided by veins visualized with SWI in healthy humans and in subjects with glioblastoma [11]. The effect of carbogen on the appearance of blood vessels is more dramatic than what would be seen with hyperoxygenation. Thus, that we saw such an effect with hyperoxygenation is very encouraging. We chose not to use hypercapnia in our study since it can lead to increased intracranial pressure [22] and carbogen is not always well tolerated.

In terms of the effect of high oxygen on cerebral perfusion in humans, the effect is variable amongst studies, ranging from 11–27% [23]; these values are similar to what has observed when caffeine is ingested [24]. High oxygen reduces perfusion, but not by very much, so in this study, we wanted to see if we could do this without having to use a material that is uncomfortable to patients. Furthermore, if perfusion drops a lot, it would have the opposite effect of oxygen.

When translating this method to humans, the difference in magnetic field strength has to be considered. Susceptibility effects will be greater with a higher magnetic field strength and with a longer TE. Since we carried out our study at higher field, we compensated by using a much shorter TE which reduces the sensitivity of our imaging to susceptibility effects. With a lower field strength as would be expected in human clinical imaging, a longer TE could be used to obtain a similar susceptibility effect to what we saw in our study. It is worth noting that studies have been carried out using susceptibility MRI at 3T which have shown good phase contrast in MS patients [25–27].

Here, we have shown that altering the inspired oxygen concentration is a viable method to identify deoxyhemoglobin-based SWI hypointensities in the EAE mouse spinal cord, and could be implemented in the clinic in MS patients. This method can be used to determine the relative contribution of deoxyhemoglobin to SWI hypointensities *in vivo* in studies imaging at multiple time points, both in EAE and in MS.

## Supporting Information

S1 Table EAE behavior scores for animals in study. (XLSX)

S2 Table Respiration and temperature data for animals in study. (XLSX)

S3 Table SWI hypointensity counting data. (XLSX)
